# MKL1 Mediates TGF-β Induced RhoJ Transcription to Promote Breast Cancer Cell Migration and Invasion

**DOI:** 10.3389/fcell.2020.00832

**Published:** 2020-08-25

**Authors:** Baoyu Chen, Yibiao Yuan, Lina Sun, Junliang Chen, Mengzhu Yang, Yongmei Yin, Yong Xu

**Affiliations:** ^1^Department of Oncology, First Hospital Affiliated to Nanjing Medical University, Nanjing, China; ^2^Key Laboratory of Targeted Intervention of Cardiovascular Disease and Collaborative Innovation Center for Cardiovascular Translational Medicine, Department of Pathophysioloy and Laboratory Center for Experimental Medicine, Nanjing Medical University, Nanjing, China; ^3^Department of Pathology and Pathophysiology, College of Life and Basic Medical Sciences, Soochow University, Suzhou, China; ^4^Department of Pathophysiology, Wuxi Medical School, Jiangnan University, Wuxi, China; ^5^Institute of Biomedical Research, Liaocheng University, Liaocheng, China

**Keywords:** transcriptional regulation, transcription factors, breast cancer metastasis, RhoJ, MKL1, ERG1

## Abstract

Differential regulation of gene transcription contributes to cancer metastasis. We investigated the involvement of a Rho GTPase (RhoJ) in breast cancer metastasis focusing on the mechanism underlying RhoJ *trans*-activation by pro-metastatic cues. We report that expression of RhoJ was up-regulated in malignant breast cancer cells compared to more benign ones. Higher RhoJ expression was also detected in human breast cancer biopsy specimens of advanced stages. RhoJ depletion attenuated breast cancer cell migration and invasion *in vitro* and metastasis *in vivo*. The pro-metastatic stimulus TGF-β activated RhoJ via megakaryocytic leukemia 1 (MKL1). MKL1 interacted with and was recruited by ETS-related gene 1 (ERG1) to the RhoJ promoter to activate transcription. In conclusion, our data delineate a novel transcriptional pathway that contributes to breast cancer metastasis. Targeting the ERG1-MKL1-RhoJ axis may be considered as a reasonable approach to treat malignant breast cancer.

## Introduction

Breast cancer is the most commonly diagnosed cancer in women worldwide. The growth in the understanding of its molecular pathogenesis notwithstanding, breast cancer remains the leading cause of cancer-related deaths in female patients claiming over 600,000 lives each year (Bray et al., 2018). The development and application of sophisticated screening techniques and personalized chemotherapies have significantly reduced the mortality rates in breast cancer patients but the long-term prognosis for those diagnosed with an advanced-stage and thus highly malignant type of breast cancer is disproportionally poor (Esteva et al., 2019). Metastatic breast cancers are characterized by aggressive behaviors of proliferation, migration, and invasion, resistance to cytotoxic chemotherapeutic drugs, and evasion of immune surveillance. Elucidation of the mechanisms whereby breast cancer cells acquire these malignant traits holds the key to novel therapeutic solutions against this malicious disease.

Accompanying the transition of a benign breast cancer cell to a more malignant one is the alteration of its transcriptome (Kwa et al., 2017). For instance, breast cancer cells, stimulated by a pro-metastatic cue (e.g., transforming growth factor), shed the expression of epithelial signature genes (e.g., CDH1 encoding E-Cadherin) and simultaneously gain the expression of mesenchymal-specific genes (e.g., VIM encoding Vimentin) in a process known as epithelial-mesenchymal transition (EMT) to facilitate migration and invasion (Bill and Christofori, 2015). In addition, transcriptional activation of pro-angiogenic factors (e.g., VEGF encoding vascular endothelial growth factor) in breast cancer cells promotes the formation of new capillaries to sustain malignant growth (Tomar et al., 2019). On the other hand, simultaneous up-regulation of anti-apoptotic genes (e.g., PD-1 encoding programmed cell death protein 1) and down-regulation of immunity priming genes (e.g., MHC II encoding major histocompatibility complex II) help breast cancers escape detection and execution by the patrolling immune cells (Wang et al., 2017).

Megakaryocytic leukemia 1 (MKL1), also known as myocardin related transcription factor A (MRTF-A), is a transcriptional modulator initially identified as a co-factor for serum response factor (SRF) responsible for activating the expression of muscle lineage-specific genes that contain the conserved CArG box within their promoters (Miano, 2015). MKL1 knockout mice are born at Mendelian ratios and exhibit no overt phenotype under physiological conditions except for a minor deficiency in mammary epithelium that renders the females unable to eject milk to nurse their offsprings (Li et al., 2006; Sun et al., 2006). Recent investigations demonstrate that MKL1 plays versatile roles in the pathogenesis of human diseases, including cardiovascular diseases, liver diseases, kidney diseases, and cancers, by regulating specific transcriptional events. For instance, Brandt et al. have shown that MKL1, in cooperation with SRF, activates integrin beta 1 (ITGB1) transcription to promote breast cancer cell dissemination (Brandt et al., 2009). Cheng et al. (2015) have reported that MKL1 mediates the *trans*-activation of metalloproteinase 9 (MMP9) in lung cancer cells to facilitate metastasis.

RhoJ belongs to the Ras superfamily of small GTP-binding proteins that exert diverse effects in a wide range of pathophysiological processes (Leszczynska et al., 2011). Of note, mounting evidence suggests that RhoJ activity is positively correlated with cell mobility (Kaur et al., 2011; Hou et al., 2013; Wilson et al., 2014; Liu et al., 2017). RhoJ transcription can be regulated by the transcription factor ERG1 in endothelial cells (Yuan et al., 2011). It remains obscure how RhoJ expression is modulated during breast cancer metastasis. Here we report that MKL1 interacts with ERG1 to activate RhoJ transcription and promote breast cancer metastasis. Therefore, targeting the ERG1-MKL1-RhoJ axis may be considered as a viable solution to treat malignant breast cancer.

## Materials and Methods

### Cell Culture and Treatment

Human breast cancer cells (MCF-7, MDA-231, MDA-468, T47D, and Hs578T) were obtained from and authenticated by the Chinese Academy of Sciences Type Culture Collection Cell Bank and were maintained in DMEM (Invitrogen). The cells were re-authenticated using a fingerprint method every 6 months in the laboratory. The last time the cells were authenticated was September 2019. Human recombinant TGF-β was purchased from R&D. Stable cells were made as previously described (Sun et al., 2013). Briefly, the cells were infected with lentivirus carrying a specific targeting siRNA or scrambled siRNA (SCR) at an MOI of 50. 48 h after infection, the cells were selected with puromycin (2.5 mg/ml) for 2 weeks.

### Plasmids and Transient Transfection

RhoJ promoter constructs and expression constructs for RhoJ, MKL1, and ERG1 have been have been previously described (Kaur et al., 2011; Yuan et al., 2011; Shi et al., 2014; Cheng et al., 2015). Small interfering RNAs were purchased from Dharmacon. Transient transfections were performed with Lipofectamine 2000 (Invitrogen). Luciferase activities were assayed 24–48 h after transfection using a luciferase reporter assay system (Promega) as previously described (Li et al., 2019d, e; Liu et al., 2019; Lu et al., 2019). Briefly, cells were plated in 12-well culture dishes (∼60,000 cells/well). The next day, equal amounts (0.1 μg) of reporter construct and effector construct were transfected into each well. DNA content was normalized by the addition of an empty vector (pcDNA3). For monitoring transfection efficiency and for normalizing luciferase activity, 0.02 μg of GFP construct was transfected into each well. Experiments were routinely performed in triplicate wells and repeated at least three times.

### Protein Extraction, Co-immunoprecipitation, and Western

Whole cell protein extraction and nuclear protein extraction were essentially performed as previously described (Shao et al., 2019; Weng et al., 2019; Yang et al., 2019a, b; Zhao et al., 2019). Specific antibodies or pre-immune IgGs (P.I.I.) were added to and incubated with cell lysates overnight before being absorbed by Protein A/G-plus Agarose beads (Santa Cruz). Precipitated immune complex was released by boiling with 1X SDS electrophoresis sample buffer. Western blot analyses were performed with commercially available antibodies: anti-RhoJ (Abcam, ab105311), anti-MKL1 (Santa Cruz, sc-32909), anti-ERG1 (Santa Cruz, sc-353), anti-FLAG (Sigma, F3165), anti-GFP (Proteintech, 50430-2), and anti-μ-actin (Sigma, A2228). Image J software was used for densitometrical quantification and densities of target proteins were normalized to those of μ-actin. Data are expressed as relative protein levels compared to the control group which is arbitrarily set as 1. All experiments were repeated at least three times.

### DNA Affinity Pull-Down

Nuclear proteins (∼100 μg) were incubated with biotin-labeled RhoJ DNA probe at room temperature for 1 h in 1× binding buffer (20 mM HEPESpH7.9, 0.1 mM EDTA, 4% glycerol, and 2 mM DTT) supplemented with BSA (50 μg per reaction), poly-dIdC, and sonicated salmon sperm DNA (100 μg per reaction). DNA-protein complexes formed were then captured by incubating with the streptavidin beads (Promega) for 1 h at 4°C on a shaking platform. Ternary complex (biotin-labeled DNA-protein-streptavidin) was washed three times with 1× binding buffer supplemented with 0.01% Triton X and 100 mM KCl for 10 min each wash. The bound proteins were eluted with 1× SDS electrophoresis sample buffer by incubating at 90°C for 10 min and analyzed by SDS-PAGE gel electrophoresis followed by Western. Experiments were repeated at least three times.

### Human Tumor Samples

Breast cancer tissues were collected, under informed consent, from surgical resection specimens of patients who had not undergone radiotherapy or chemotherapy in the First Affiliated Hospital of Nanjing Medical University following the guidelines of the intramural Committee on Human Studies as previously described (Sun et al., 2013). Tumor differentiation was graded using the Edmondson grading system. For Immunohistochemical staining, paraffin sections were dewaxed and heated in EDTA repairing buffer (pH 9.0) for 15 min for antigen retrieval. The sections then were blocked with 5% BSA and incubated with anti-RhoJ (1:100, Sigma, HPA003050) overnight at 4°C. The next day, the slides were incubated with an HRP-conjugated secondary antibody for 30 min and developed for 5 min using diaminobenzidine (DAB) as the substrate. Images were visualized and captured by an Olympus IX-70 microscope. Scoring (high vs. low staining) was performed by two pathologists in a blinded fashion.

### RNA Extraction, and Real-Time PCR

RNA was extracted with the RNeasy RNA isolation kit (Qiagen). Reverse transcriptase reactions were performed using a SuperScript First-strand Synthesis System (Invitrogen). Real-time PCR reactions were performed on an ABI Prism Stepone Plus system. *C*t values of target genes were normalized to the *C*t values of 18rRNA using the ΔΔ*C*t method and expressed as relative mRNA expression levels compared to the control group which is arbitrarily set as 1.

### Chromatin Immunoprecipitation (ChIP) and Re-ChIP

Chromatin immunoprecipitation assays were performed essentially as described before (Li et al., 2018a,b,c,d,e, 2019a,b,c; Liu et al., 2018; Yang et al., 2018; Zeng et al., 2018; Zhang et al., 2018a,b; Fan et al., 2019; Kong et al., 2019a, b). Briefly, chromatin was cross-linked with 1% formaldehyde for 8 min room temperature, and then sequentially washed with ice-cold phosphate-buffered saline, Solution I (10 mM HEPES, pH 7.5, 10 mM EDTA, 0.5 mM EGTA, 0.75% Triton X-100), and Solution II (10 mM HEPES, pH 7.5, 200 mM NaCl, 1 mM EDTA, 0.5 mM EGTA). Cells were incubated in lysis buffer (150 mM NaCl, 25 mM Tris pH 7.5, 1% Triton X-100, 0.1% SDS, 0.5% deoxycholate) supplemented with protease inhibitor tablet. DNA was fragmented into 500 bp pieces using a Branson 250 sonicator. Aliquots of lysates containing 100 μg of protein were used for each immunoprecipitation reaction with the following antibodies: anti-MKL1 (Santa Cruz, sc-32909), anti-ERG1 (Santa Cruz, sc-353), or pre-immune IgG followed by adsorption to protein A/G PLUS-agarose beads (Santa Cruz Biotechnology). Precipitated DNA-protein complexes were washed sequentially with RIPA buffer (50 mM Tris, pH 8.0, 150 mM NaCl, 0.1% SDS, 0.5% deoxycholate, 1% Nonidet P-40, 1 mM EDTA), high salt buffer (50 mM Tris, pH 8.0, 500 mM NaCl, 0.1% SDS, 0.5% deoxycholate, 1% Nonidet P-40, 1 mM EDTA), LiCl buffer (50 mM Tris, pH 8.0, 250 mM LiCl, 0.1% SDS, 0.5% deoxycholate, 1% Nonidet P-40, 1 mM EDTA), and TE buffer (10 mM Tris, 1 mM EDTA pH 8.0), respectively. DNA-protein cross-link was reversed by heating the samples to 65°C overnight. Proteins were digested with proteinase K (Sigma), and DNA was phenol/chloroform-extracted and precipitated by 100% ethanol. Precipitated genomic DNA was amplified by real-time PCR with primers spanning the human RhoJ gene promoters. A total of 10% of the starting material is also included as the input. Data are then normalized to the input and expressed as fold changes compared to the control group.

### Scratch-Wound Healing/Migration Assay

Cell migration assay has been described previously (Yang et al., 2019a). Cells were re-suspended in serum-free media. When the cells reached confluence, scratch wound was created by using a sterile micropipette tip. Cell migration was calculated by Image Pro. Data were expressed as % migration compared to control arbitrarily set as 100%.

### Boyden Chamber Assay

24-well inserts (Costar) with 10 μg/ml Matrigel (Sigma) were used for invasion assays (for migration assay, no matrigel was added). Cells were re-suspended in serum-free media with or without TGF-β and plated into the upper chamber with the lower chamber filled with complete media. Following exposure to TGF-β, the cells on the upper chamber were removed. Invaded cells were stained with 0.1% crystal violet and counted at 200× magnification in 10 different fields. Experiments were repeated three times. Data were expressed as relative migration/invasion compared to control arbitrarily set as 100%.

### Heterotopic Xenographt

All animal studies were performed under the guidelines of the Nanjing Medical University Intramural Ethic Committee on Humane Treatment of Experimental Animals. Anesthetized 6- to 8-week-old SCID mice were injected subcutaneously via the flank with, per mouse, 5 × 10^6^ cells in phosphate-buffered saline. The mice were sacrificed 3 weeks after implantation and tumors were dissected from the mice and weighed. Tumor volume was calculated according to the following formula: 0.5 × length × width^2^.

### *In vivo* Metastasis

Anesthetized 6- to 8-week-old SCID mice were randomly divided into different groups and injected via tail vein with MCF cells (1 × 10^6^ per mouse, via tail vein). 25 days following injection, mice were sacrificed and metastasized nodules in the lungs were counted. All animal experiments were performed double-blindly.

### Statistical Analysis

Two-sided *T*-test (for experiments involving two groups) or one-way ANOVA with *post hoc* Scheffe analyses (for experiments involving with at least three groups) were performed using an SPSS package. *p*-values smaller than 0.05 were considered statistically significant (^∗^).

## Results

### RhoJ Expression Is Up-Regulated in Malignant Types of Breast Cancers

**FIGURE 1:**
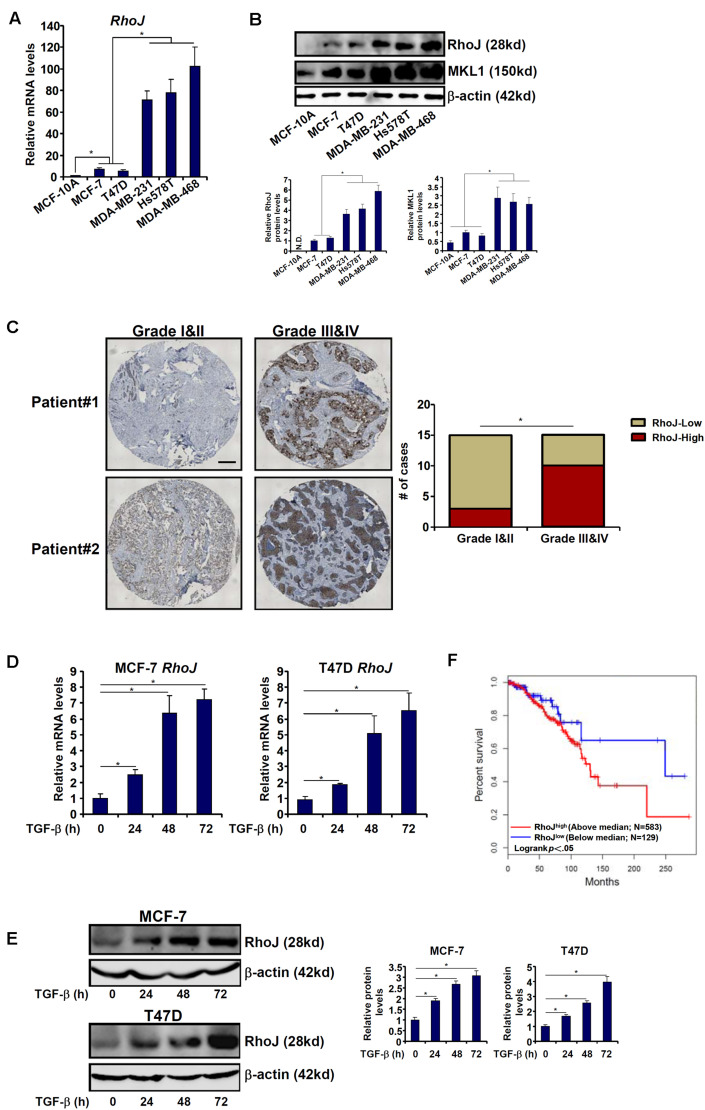
RhoJ expression correlates with breast cancer malignancy. **(A,B)** Expression of Rho family members in different breast cancer cell lines was assessed by qPCR **(A)** and Western blotting **(B)**. **(C)** Human breast tumor samples were processed as described in Section “Materials and Methods.” RhoJ expression was examined by immunohistochemistry. **(D,E)** MCF-7 and T47D cells were treated with TGF-β (2 ng/ml) and harvested at indicated time points. Expression levels of RhoJ were examined by qPCR **(D)** and Western **(E)**. **(F)** Kaplan–Meier plot of overall survival in patients with high and low RhoJ expression (median RhoJ expression as the cut-off). Asterisk indicates *p* value smaller than 0.05.

We first compared the levels of RhoJ among normal mammary epithelial cells (MCF-10A) and breast cancer cells of varying malignancies (three with strong metastatic capability, MDA-231, MDA-468, and Hs578T, and two with lesser aggressive behavior, MCF-7 and T47D). RhoJ expression was progressively up-regulated, at both mRNA (Figure 1A) and protein (Figure 1B) levels, as normal mammary epithelial cells transitioned to breast cancer cells. To define a broad role for RhoJ in breast cancer metastasis, we then examined the expression levels of different Rho GTPases in different breast cancer cell lines. Out of 19 different Rho GTPases tested, RhoJ expression showed the most significant correlation with breast cancer cell malignancy (Supplementary Figure S1). In addition, we observed in patients with advanced stages of breast cancer elevated RhoJ mRNA expression compared to patients with a less malignant phenotype (Figure 1C).

Next, we assessed RhoJ expression levels in response to different pro-malignancy stimuli. Treatment with TGF-β significantly increased RhoJ expression in a time course-dependent manner (Figures 1D,E) in both MCF-7 and T47D cells. By comparison, activation of the canonical Wnt signaling pathway, achieved by the addition of either a CK1 inhibitor (Supplementary Figure S2A) or a GSK3 inhibitor (Supplementary Figure S2B) or over-expression of a constitutively active β-catenin (Supplementary Figure S2C), failed to augment RhoJ expression (the β-catenin target Axin2 was included as a positive control).

We then attempted to correlate RhoJ expression with breast cancer malignancies and prognosis using the datasets disposed in the database^1^. Kaplan–Meier analysis revealed that high RhoJ expression was associated with poorer survival in breast cancer patients (Figure 1F). Together, these data suggest that RhoJ expression is intimately associated with breast cancer malignancy *in vitro* and *in vivo*.

### RhoJ Potentiates Breast Cancer Cell Migration and Invasion

**FIGURE 2:**
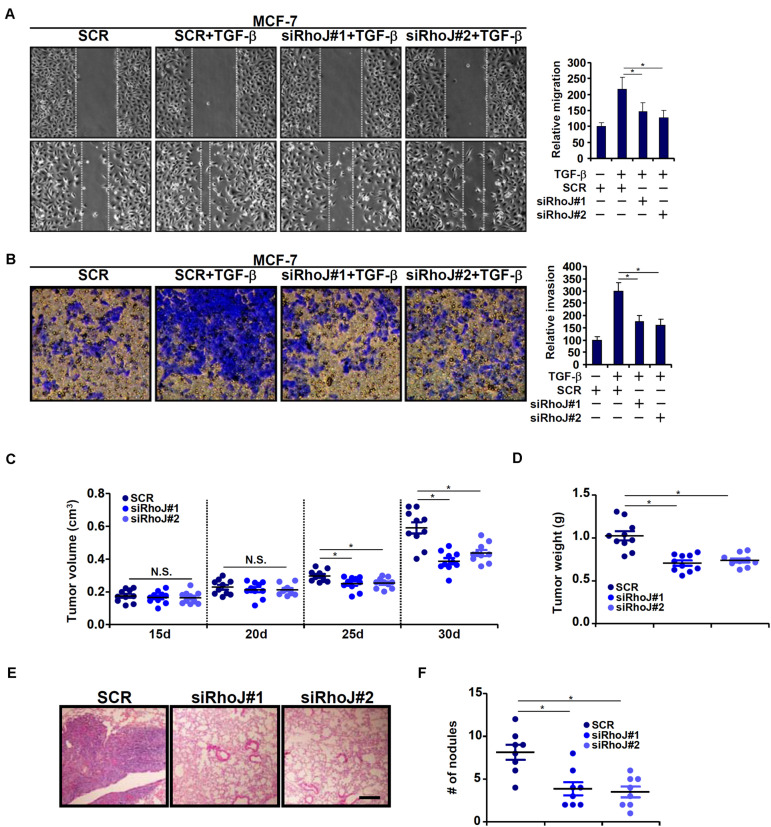
RhoJ potentiates breast cancer cell migration and invasion. **(A,B)** Stable MCF-7 cells were treated with TGF-β (2 ng/ml) for 48 h. Cell migration **(A)** and invasion **(B)** were evaluated as described under Section “Materials and Methods.” **(C,D)** Heterotopic xenographt assay was performed as described in Section “Materials and Methods.” *N* = 10 mice for each group. **(E,F)** *In vivo* metastasis was performed as described in Section “Materials and Methods.” *N* = 8 mice for each group. Asterisk indicates *p* value smaller than 0.05.

We next evaluated the effect of RhoJ on breast cancer cell migration and invasion. To this end, breast cancer cells were infected with lentivirus carrying siRNA targeting RhoJ or scrambled siRNA (SCR) and selected with puromycin for 2 weeks to knock down endogenous RhoJ (Supplementary Figure S3 for knockdown efficiencies). RhoJ knockdown significantly dampened TGF-β induced migration and invasion of MCF-7 (Figures 2A,B) and T47D (Supplementary Figures S4A,B) cells, as assessed by wound healing assay and Boyden chamber transwell assay, respectively. RhoJ knockdown also suppressed basal levels of migration and invasion in highly malignant MDA-231 (Supplementary Figures S4C,D), Hs578T (Supplementary Figures S4E,F), and MDA-468 (Supplementary Figures S4G,H) cells.

Next, two different animal models were exploited to evaluate the effect of RhoJ knockdown on breast cancer cell migration/invasion *in vivo*. In the first model, stable MCF-7 cells were inoculated subcutaneously into the nude mice. As shown in Figure 2C, although the amplification of tumor volume was not altered by RhoJ knockdown at earlier points (15 and 20 days) following the inoculation, it was significantly slowed toward the end (25 and 30 days). Consistently, when the mice were sacrificed it was discovered that tumor weight was significantly smaller in mice receiving the inoculation of RhoJ-depleted cells than the control cells (Figure 2D). In the second model, the cells were injected into the tail veins and the mice were sacrificed 5 weeks later to evaluate the formation of tumorous nodules in the lungs. RhoJ silencing again significantly suppressed the metastatic abilities of breast cancer cells (Figures 2E,F).

### MKL1 Activates RhoJ Expression in Breast Cancer Cells

**FIGURE 3:**
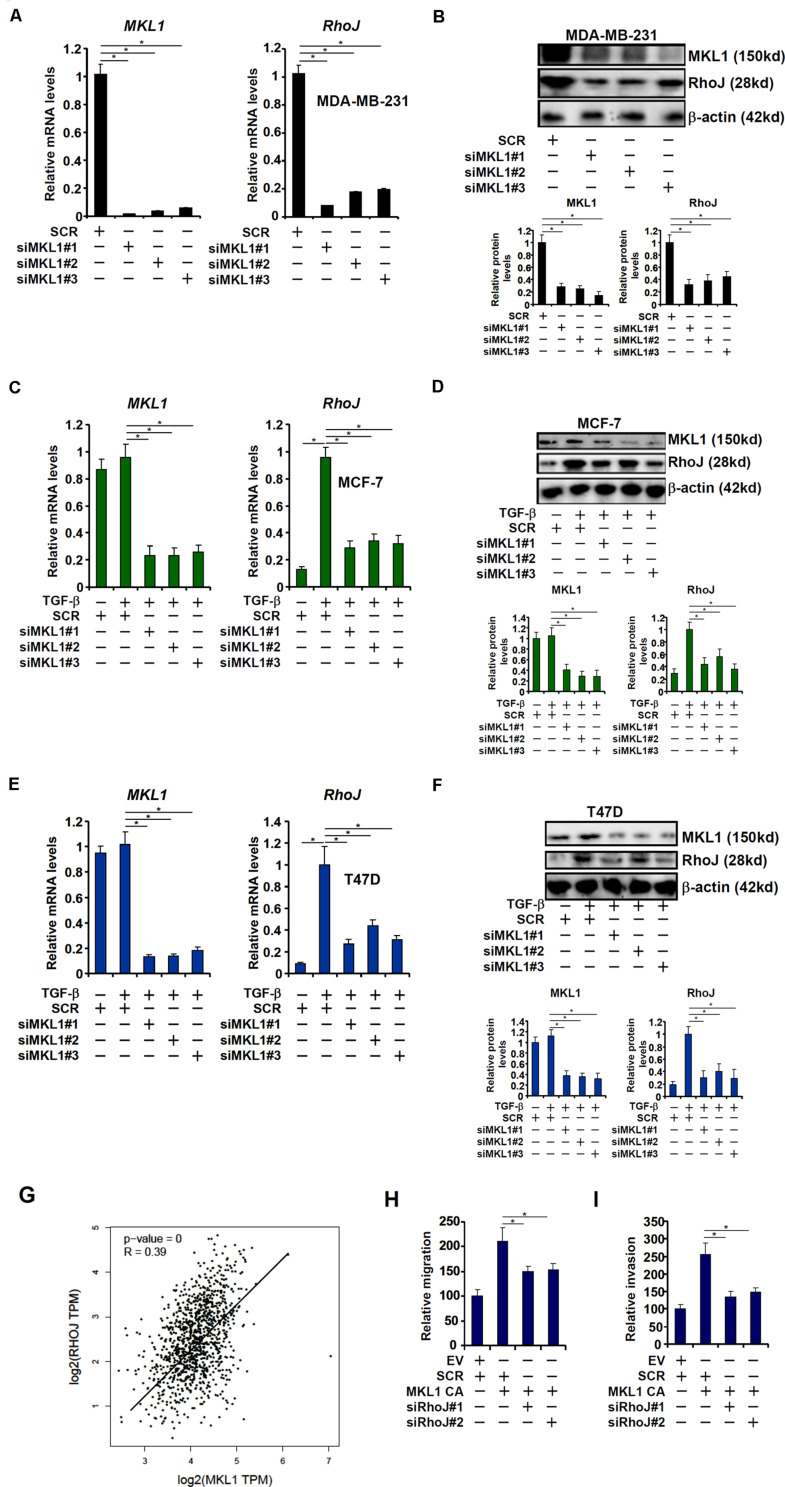
MKL1 activates RhoJ expression in breast cancer cells. **(A,B)** Gene expression in stable MDA-MB-231 cells was examined by qPCR and Western. **(C,D)** Stable MCF-7 cells were treated with or without TGF-β for 48 h. Gene expression was examined by qPCR and Western. **(E,F)** Stable T47D cells were treated with or without TGF-β for 48 h. Gene expression was examined by qPCR and Western. **(G)** Expression data of MKL1 and RhoJ were extracted from the public database to draw the scatter plot. Pearson correlation co-efficient was calculated. **(H,I)** MCF-7 cells were transfected with a constitutively active (CA) MKL1 or an empty vector (EV) in the presence or absence of siRNA targeting RhoJ. Cell migration **(H)** and invasion **(I)** were evaluated as described under Section “Materials and Methods” and quantified by Image Pro. Asterisk indicates *p* value smaller than 0.05.

The transcriptional modulator MKL1 has been shown to promote breast cancer metastasis (Brandt et al., 2009). A microarray-based screen, which was unrelated to the current report and aimed to identify novel MKL1 target genes in cardiomyocyte, revealed that RhoJ expression might be regulated by MKL1 (Xu Y, unpublished data). To this end, MKL1 was stably knocked down using a similar strategy as RhoJ. Depletion of MKL1 decreased basal RhoJ mRNA levels in highly metastatic cells MDA-231 (Figures 3A,B). By comparison, the loss of MKL1 did not significantly alter the levels of other Rho GTPases (Supplementary Figure S5). MKL1 knockdown also abolished TGF-β induced RhoJ expression in poorly metastatic cells MCF-7 (Figures 3C,D) and T47D (Figures 3E,F). Of note, there was a significant correlation between MKL1 and RhoJ expression levels in human breast cancer tissues (Figure 3G). Enhanced migration and invasion (Figures 3H,I and Supplementary Figures S6A,B) of MCF-7 cells driven by MKL1 over-expression was blunted by RhoJ silencing. Congruently, RhoJ over-expression partially rescued the loss of migratory (Supplementary Figure S6C) and invasive (Supplementary Figure S6D) abilities of MDA-231 cells following MKL1 depletion. Together, these data suggest that MKL1 might contribute to breast cancer cell migration/invasion by modulating RhoJ expression.

### MKL1 Directly Regulates RhoJ Transcription in Breast Cancer Cells

**FIGURE 4:**
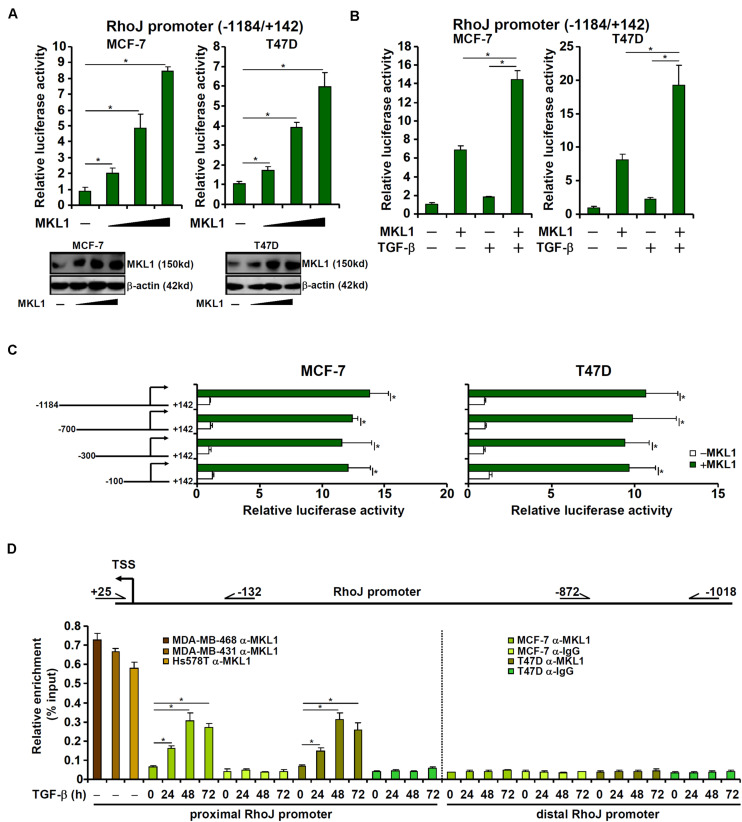
MKL1 directly regulates RhoJ transcription in breast cancer cells. **(A)** A RhoJ promoter-luciferase construct was transfected into MCF-7 and T47D cells with or without MKL1. Luciferase activities were normalized by protein concentration and GFP fluorescence. Data are expressed relative luciferase activities compared to the control group. Inset, MKL1 expression levels in different groups were examined by Western blotting. **(B)** A RhoJ promoter-luciferase construct was transfected into MCF-7 and T47D cells with or without MKL1 followed by treatment for TGF-β 48 h. Luciferase activities were normalized by protein concentration and GFP fluorescence. Data are expressed relative luciferase activities compared to the control group. **(C)** RhoJ promoter-luciferase constructs of different lengths were transfected into MCF-7 and T47D cells with or without MKL1. Luciferase activities were normalized by protein concentration and GFP fluorescence. Data are expressed relative luciferase activities compared to the control group. **(D)** ChIP assays were performed with anti-MKL1 or IgG using nuclear lysates isolated from the cells as indicated. Asterisk indicates *p* value smaller than 0.05.

MKL1 over-expression augmented the activity of a RhoJ promoter (−1184/+142) construct in a dose-dependent manner (Figure 4A), suggesting that activation of RhoJ by MKL1 likely occurred at the transcription level. MKL1 over-expression also amplified the induction of RhoJ transcription by TGF-β (Figure 4B). Of note, MKL2, a closely related family member of MKL1, failed to activate RhoJ transcription in reporter assays (Supplementary Figure S7A). Interference of MKL1 activity by either shRNA (Supplementary Figure S7B) or dominant negative (DN) mutation (Supplementary Figure S7C) or treatment with a small-molecule MKL1 inhibitor CCG-1423 (Supplementary Figure S7D) abrogated the induction of RhoJ transcription by TGF-β.

Next, serially deleted RhoJ promoter constructs were transfected into cells with or without MKL1. As shown in Figure 4C, over-expression of MKL1 activated the RhoJ promoter construct even when deletion extended to −100 relative to the transcription start site. ChIP assays confirmed that robust basal MKL1 binding on this region (−132/+25), but not a distal region (−1018/−872), of the RhoJ promoter was detectable in the highly malignant cells; MKL1 binding at basal conditions was relatively weak in lesser malignant cells but could be strongly stimulated by TGF-β treatment (Figure 4D). Together, these data suggest that aggressive behavior of breast cancer cells might be, at least in part, attributable to MKL1-mediated RhoJ *trans*-activation.

### MKL1 Is Recruited by ERG1 to Activate RhoJ Transcription

**FIGURE 5:**
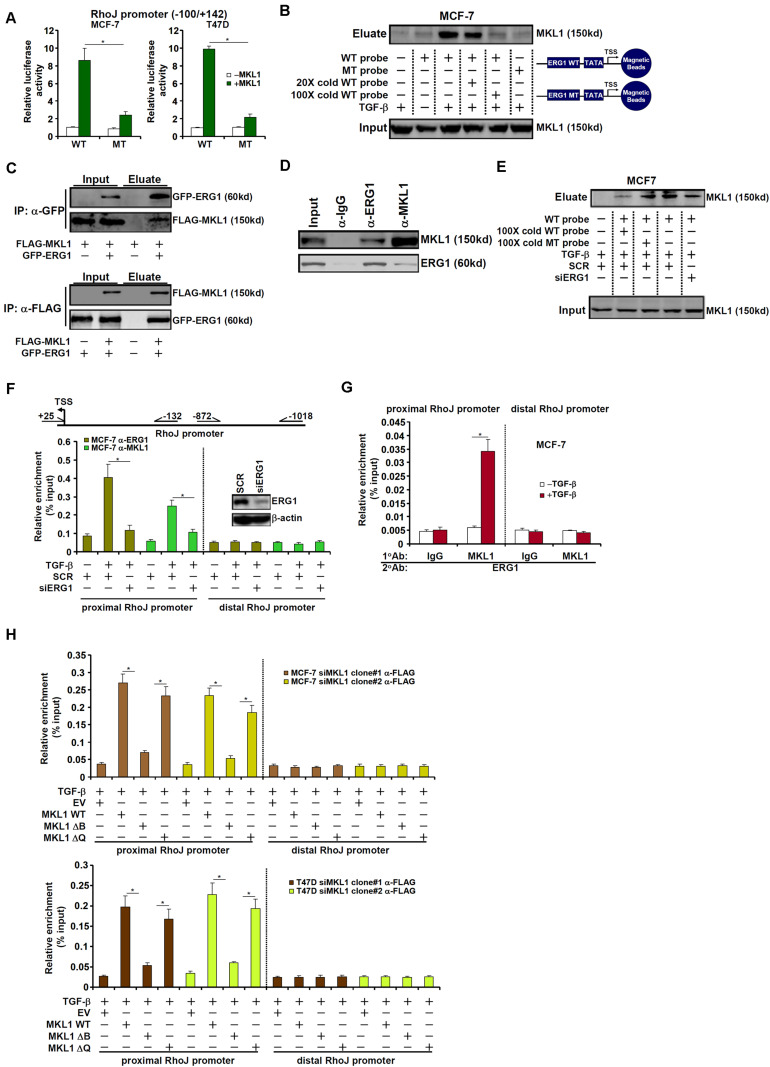
MKL1 is recruited by ERG1 to activate RhoJ transcription. **(A)** Wild type or mutant RhoJ promoter construct was transfected into MCF-7 and T47D cells with or without MKL1. Luciferase activities were normalized by protein concentration and GFP fluorescence. Data are expressed relative luciferase activities compared to the control group. **(B)** MCF-7 cells were treated with or without TGF-β for 24 h. Nuclear proteins were extracted and DNA affinity pull-down assay was performed. **(C)** HEK293 cells were transfected with tagged expression constructs. Immunoprecipitation was performed with indicated antibodies. **(D)** MCF-7 cells were treated with TGF-β for 24 h. Nuclear lysates were extracted and immunoprecipitation was performed with indicated antibodies. **(E,F)** MCF-7 cells were transfected with indicated siRNAs followed by treatment with TGF-β for 24 h. Nuclear proteins were extracted and DNA affinity pull-down assay and ChIP assay were performed. **(G)** MCF-7 cells were treated with or without TGF-β for 24 h. Nuclear proteins were extracted and Re-ChIP assay was performed with indicated antibodies. **(H)** Stable MKL1 KD MCF-7 and T47D cells were transfected with FLAG-tagged MKL1 expression constructs or an empty vector (EV) followed by treatment with TGF-β for 24 h. Nuclear proteins were extracted and ChIP assay was performed. Asterisk indicates *p* value smaller than 0.05.

Being a co-factor, MKL1 relies on the recruitment by sequence-specific transcription factors to bind to DNA (Yu et al., 2014; Fan et al., 2015; Weng et al., 2015; Li et al., 2018c). The minimal RhoJ promoter construct (−100/+142) that responds to MKL1 over-expression contains a conserved motif for ETS-related gene 1 (ERG1) (Yuan et al., 2011); mutation of this motif abrogated promoter activation by MKL1 over-expression (Figure 5A). DNA affinity pull-down experiments showed that MKL1 bound to the wild type, but not the ERG1 site mutated, DNA probe containing the proximal RhoJ promoter (Figure 5B). Co-immunoprecipitation experiments confirmed an interaction between MKL1 and ERG1 (Figures 5C,D). Further analyses revealed that the N-terminal basic (B) domain of MRTF-A mediated its interaction with ERG1 (Supplementary Figure S8A). Several lines of evidence allude to a dynamic interplay between MKL1 and ERG1 in regulating RhoJ transcription: (1) co-expression of MKL1 and ERG1 synergistically activated the RhoJ promoter (Supplementary Figure S8B) while MKL1 DN (Supplementary Figure S8C) or CCG-1423 (Supplementary Figure S8D) treatment suppressed RhoJ promoter activity in the presence of ERG1; (2) siRNA-mediated silencing of ERG1 not only abrogated the recruitment of MKL1 to the RhoJ promoter, as assessed by DNA affinity pull-down assay (Figure 5E and Supplementary Figure S8E) and ChIP assay (Figure 5F and Supplementary Figure S8F), but blunted activation of the RhoJ promote by MKL1 (Supplementary Figure S8G); (3) TGF-β promoted the formation of an ERG1-MKL1 complex on the same site in MCF-7 and T47D cells as evidenced by Re-ChIP assay (Figure 5G and Supplementary Figure S8H); (4) DNA affinity pull-down (Supplementary Figure S8I) and ChIP (Figure 5H) assays suggested that the MKL1 mutant that lacks the basic domain and hence is unable to interact with ERG1 failed to be recruited to the RhoJ promoter. Combined, these data suggest that an ERG1-MKL1 complex activates RhoJ transcription in breast cancer cells.

## Discussion

The ascendency of the transcriptomic era has not only greatly broadened the understanding of cancer pathogenesis but enabled identification of druggable targets to treat malignant cancer (Brower, 2011; Dawson and Kouzarides, 2012). In the present study we delineate a novel pathway wherein MKL1 regulates breast cancer metastasis through activating RhoJ transcription. Although MKL1 is dispensable for embryonic development, recent studies have implicated MKL1 in the pathogenesis of a host of human diseases. Specifically, MKL1 has been linked to the oncogenesis of hepatocellular carcinoma (Muehlich et al., 2012; Hermanns et al., 2017), lymphoma (Bjorkholm et al., 2013), lung carcinoma (Cheng et al., 2015), ovarian carcinoma (Xu et al., 2017), thyroid carcinoma (Cheng et al., 2019), colorectal carcinoma (Werner et al., 2019), and breast carcinoma (Brandt et al., 2009; Xiang et al., 2017). MKL1 executes its pro-oncogenic and pro-metastatic activities primarily by orchestrating specific transcriptional events. For instance, MKL1-mediated transcriptional activation of a slew of integrin (ITGA) genes serves as an integral step in focal adhesion kinase (FAK) signaling to facilitate cancer cell spreading (Kishi et al., 2016). Alternatively, activation of metalloproteinase (MMP) transcription by MKL1 contributes to degradation of the extracellular matrix and cancer metastasis (Cheng et al., 2015, 2019; Xu et al., 2017). MKL1 can also activate the transcription of deleted in liver cancer (DLC1) and myoferlin to defy oncogenic senescence and preserve cancer cell viability (Hampl et al., 2013; Hermanns et al., 2017). Samaeekia et al. (2017) have reported that MKL1 may contribute to the maintenance of cancer cell stemness by activating interleukin 11 (IL11) transcription. Whitson et al. have recently reported that MKL1 directs a transcriptional program downstream of the non-canonical Hedgehog pathway in basal cell carcinoma to aid drug resistance (Whitson et al., 2018). Our data add to the mountain of evidence that MKL1 integrates transcriptional events to skew cancer cell phenotype to a more malignant type.

MKL1 is a co-factor relying on its interaction with sequence-specific transcription factors to participate in transcriptional regulation. ERG1 is one of the best characterized transcription factors that regulate RhoJ transcription. First reported by Yuan et al. (2011), ERG1 binds to the most proximal region (−30/−23 relative to the transcription start site) of RhoJ promoter and activates RhoJ transcription in vascular endothelial cells. This finding was further validated by a recent single-cell based transcriptomic study in human hematoendothelial cells (Angelos et al., 2018). Here we provide evidence to show that ERG1 is a novel binding partner for MKL1. It remains to be determined whether the functional interplay between MKL1 and ERG1 converges solely on RhoJ, but there is evidence to suggest that there are potentially several oncogenesis-related events co-regulated by MKL1 and ERG1. For instance, it has been reported that both ERG1 (Zhang et al., 2020) and MKL1 (Bernard et al., 2015) can skew cellular metabolism to favor glycolysis leading to hyperproliferation of cells. MKL1 (Morita et al., 2007; Xiang et al., 2017) and ERG1 (Kao et al., 2014; Mochmann et al., 2014) can both regulate cancer cell metastasis by promoting epithelial-mesenchymal transition (EMT). MKL1 (Evelyn et al., 2016) and ERG1 (Sreenath et al., 2017) are separately involved in regulation of ER stress-related transcription, a process key to oncogenesis. These scattered pieces of evidence argue that there might be a larger-than-expected overlap of target genes for ERG1 and MKL1 genome wide that contribute to breast cancer metastasis. Future studies employing ChIP-seq techniques would help clarify this lingering issue.

Despite the uncovering of a new transcriptional complex (MKL1-ERG1) that mediates RhoJ transcription in breast cancer cells, major limitations dampen the translational impact of our study. First, we have used the subcutaneous implantation model and the tail veil injection model to evaluate breast cancer metastasis; neither model is ideal for the assessment of how primary tumor cells migrate away and spread to distal sites, a critical part of the metastatic process. Future studies should exploit more clinically relevant metastatic models (Holen et al., 2017) to determine whether this ERG1-MKL1-RhoJ axis can contribute to breast cancer metastasis *in vivo*. Second, pathobiological functions of RhoJ are not only determined by its overall expression levels but its activity. For instance, RhoJ activity, as measured by its GTP-bound form and the phosphorylation of its downstream kinases, is up-regulated during vascular morphogenesis (Yuan et al., 2011). Of note, Gao et al. (2012) have reported that MKL1 can regulate the expression of GEF-H1, a guanine nucleotide exchange factor, to modulate RhoA activity in megakaryocytes. Whether TGF-β stimulation could enhance RhoJ activity, in addition to boosting its expression, and whether MKL1 plays a role in regulating RhoJ activity presumably by altering the expression of GEFs remain to be investigated. Third, we examined the effect of RhoJ expression on over-all survival, but not metastasis-free survival, for breast cancer patients. In addition, the human data are not vigorously verified in multiple cohorts. Clearly, these limitations severely compromise the conclusiveness of our study and need to be addressed in future studies.

In summary, we provide evidence to implicate the ERG1-MKL1 axis in RhoJ *trans*-activation and breast cancer metastasis. Small-molecule compounds for some components of this axis are available and proven effective in certain cell and animal models. Based on our observation as reported here, these chemicals hopefully can be considered as a potential treatment option for the most malignant forms of breast cancer.

## Data Availability Statement

The raw data supporting the conclusions of this article will be made available by the authors, without undue reservation, to any qualified researcher.

## Ethics Statement

The studies involving human participants were reviewed and approved by Nanjing Medical University Committee on Human Studies. The patients/participants provided their written informed consent to participate in this study. The animal study was reviewed and approved by Nanjing Medical University Intramural Ethic Committee on Humane Treatment of Experimental Animals.

## Author Contributions

YX conceived the project. BC, YiY, LS, JC, and MY performed experiments and collected the data. JC, LS, and YoY handled funding. YoY provided supervision. YX wrote the manuscript. All authors designed experiments and analyzed the data.

## Conflict of Interest

The authors declare that the research was conducted in the absence of any commercial or financial relationships that could be construed as a potential conflict of interest.

## Footnotes

1 http://gepia.cancer-pku.cn/

## Supplementary Material

Ensure that any supplementary material is correctly published at this link: https://www.frontiersin.org/articles/10.3389/fcell.2020.00832/full#supplementary-material
