# Supplementary material for: MKL1 Mediates TGF-β Induced RhoJ Transcription to Promote Breast Cancer Cell Migration and Invasion
Source: Front Cell Dev Biol. 2020 Aug 25;8:832. doi: 10.3389/fcell.2020.00832 (PMC7478007; doi:10.3389/fcell.2020.00832)
Supplement: Supplementary file 1 [file Table_1.doc]

**Sun LN *et al*: MKL1 mediates TGF-β induced RhoJ transcription to promote breast cancer metastasis**

**Online supplementary material**

**Supplementary Figures:**

**Fig.S1**: Expression levels of Rho GTPases in different breast cancer cells were examined by qPCR.

**Fig.S2**: (**A**) MCF-7 cells were treated with increasing concentrations (1-10μM) of a specific CK1 inhibitor D4476 for 24 hours. RhoJ levels were examined by Western. (**B**) MCF-7 cells were treated with increasing concentrations (5-50μM) of a specific GSK3 inhibitor SB216763 for 24 hours. RhoJ levels were examined by Western. (**C**) MCF-7 cells were transfected with a constitutively active β-catenin or an empty vector (EV). RhoJ levels were examined by Western.

**Fig.S3**: RhoJ knockdown efficiencies in different stable breast cancer cells were examined by Western.

**Fig.S4**: (**A, B**) Stable T47D cells were treated with TGF-β (2ng/ml) for 48h. Cell migration (A) and invasion (B) were evaluated as described under *Methods*. (**C, D**) Stable MDA-231 cell migration (C) and invasion (D) were evaluated as described under *Methods*. (**E, F**) Stable Hs578T cell migration (E) and invasion (F) were evaluated as described under *Methods*. (**G, H**) Stable MDA-468 cell migration (G) and invasion (H) were evaluated as described under *Methods*.

**Fig.S5**: MDA-231 cells were transfected with siRNA targeting MKL1 or scrambled siRNA (SCR). Expression levels of small GTPases were examined by qPCR.

**Fig.S6**: (**A, B**) MCF-7 cells were transfected with a constitutively active (CA) MKL1 or an empty vector (EV) in the presence or absence of siRNA targeting RhoJ. Cell migration (A) and invasion (B) were evaluated as described under *Methods*. (**C, D**) Stable MDA-231 cells were transfected with RhoJ or an empty vector (EV). Cell migration (C) and invasion (D) were evaluated as described under *Methods*.

**Fig.S7**: (**A**) A RhoJ promoter construct was transfected into MCF7 cells with increasing amount of MKL1 or MKL2. Data are expressed relative luciferase activities compared to the control group. (**B**) A RhoJ promoter construct was transfected into MCF7 cells with MKL1 shRNA plasmid followed by TGF-β treatment. Data are expressed relative luciferase activities compared to the control group. (**C**) A RhoJ promoter construct was transfected into MCF7 cells with MKL1 DN followed by TGF-β treatment. (**D**) A RhoJ promoter construct was transfected into MCF7 cells followed by TGF-β or CCG-1423 (5μM, 10μM) treatment.

**Fig.S8**: (**A**) Different FLAG-tagged MKL1 expression constructs were transfected into HEK293 cells along with GFP-tagged ERG1. Immunoprecipitation was performed with anti-FLAG. (**B**) A RhoJ promoter construct was transfected into MCF7 cells with ERG1 and increasing doses of MKL1. Data are expressed relative luciferase activities compared to the control group. (**C**) A RhoJ promoter construct was transfected into MCF7 cells with ERG1 and increasing doses of MKL1 DN. Data are expressed relative luciferase activities compared to the control group. (**D**) A RhoJ promoter construct was transfected into MCF7 cells with ERG1 and increasing doses of CCG-1423 (5μM, 10μM). Data are expressed relative luciferase activities compared to the control group. (**E, F**) T47D cells were transfected with indicated siRNAs followed by treatment with TGF-β for 24h. Nuclear proteins were extracted and DNA affinity pull-down assay and ChIP assay were performed. (**G**) A RhoJ promoter construct was transfected into MCF7 and T47D cells with MKL1 in the presence or absence of ERG1 siRNA. Data are expressed relative luciferase activities compared to the control group. (**H**) T47D cells were treated with or without TGF-β for 24h. Nuclear proteins were extracted and Re-ChIP assay was performed with indicated antibodies. (**I**) Stable MKL1 KD MCF-7 and T47D cells were transfected with FLAG-tagged MKL1 expression constructs or an empty vector (EV) followed by treatment with TGF-β for 24h. Nuclear proteins were extracted and DNA affinity pull-down assay was performed.
